# Genetic variation in *IGF1* predicts renal cell carcinoma susceptibility and prognosis in Chinese population

**DOI:** 10.1038/srep39014

**Published:** 2016-12-15

**Authors:** Qiang Cao, Chao Liang, Jianxin Xue, Pu Li, Jie Li, Meilin Wang, Zhengdong Zhang, Chao Qin, Qiang Lu, Lixin Hua, Pengfei Shao, Zengjun Wang

**Affiliations:** 1 Department of Urology, the First Affiliated Hospital of Nanjing Medical University, Nanjing, Jiangsu 210029, China; 2 Department of Molecular & Genetic Toxicology, Nanjing Medical University, Nanjing, Jiangsu 210029, China

## Abstract

Insulin-like growth factor 1 (IGF1) and IGF binding protein 3 (IGFBP3) play an important role in the development and progression of renal cell carcinoma (RCC). We evaluated the association of functional polymorphisms in *IGF1* and *IGFBP3* with susceptibility and prognosis of RCC. We genotyped nine potentially functional polymorphisms in *IGF1* and *IGFBP3* and assessed their association with risk of RCC in a two-stage case-control study compromising 1027 cases and 1094 controls, and with prognosis in a cohort of 311 patients. We found rs5742714 in the 3′-UTR of *IGF1* was significantly associated with risk and prognosis of RCC. In the combined set, the rs5742714 GC/CC genotypes were significantly associated with decreased risk of RCC compared with the GG genotype (OR = 0.82; 95% CI = 0.68–0.98, *P* = 0.002). Furthermore, patients with the rs5742714 GC/CC genotypes showed improved survival than those with the GG genotype (Log-rank *P* = 0.025, HR = 0.36, 95% CI = 0.14–0.93). Besides, the rs5742714 GC/CC genotypes were associated with significantly decreased expression of *IGF1* mRNA and lower IGF1 serum levels. Moreover, the luciferase reporter assays revealed the potential effect of rs5742714 genotype on the binding of microRNAs to *IGF1*. Our findings suggest that the *IGF1* polymorphism rs5742714 may be a genetic predictor of susceptibility and prognosis of RCC.

Renal cell carcinoma (RCC) is the predominant form of kidney malignancy, accounting for more than 80% of all malignant kidney tumors^1^,^2^. Approximately 25% of patients show developed metastases at diagnosis and it is likely that 30% of the remaining patients will develop metastases even following surgical treatment^3^,^4^. Patient prognosis is generally poor following metastasis^3^. Currently, the prediction of RCC prognosis largely depends on conventional prognostic factors such as pathological tumor stage and grade.

In recent years, different genetic variations have been identified and associated with the development and prognosis of RCC in several candidate gene studies^5^,^6^,^7^,^8^ as well as a large genome-wide association study^9^. For instance, genetic polymorphisms in angiogenesis-related genes, which play a crucial role in the pathogenesis of RCC, were proved susceptibility loci or prognostic predictors of RCC^5^,^7^. Our previous studies have found that polymorphisms at the *VHL* and *HIF1A* genes could jointly influence RCC progression and survival^6^. However, these findings were not consistent across studies in different populations, which may indicate differences in the genetic architecture of ethnic groups, and investigating genetic variations in other candidate genes is still required.

The insulin-like growth factor (IGF) system plays a crucial role in regulating cell proliferation, differentiation, and apoptosis^10^,^11^. The system consists of IGF1 and IGF2 as well as their cell surface receptors (IGF1R and IGF2R), and six specific IGF-binding proteins (IGFBPs). IGFBP3, which is the predominant IGFBP, binds to the majority of circulating IGF1 to regulate its biological activity. Aberrant expression or regulation of *IGF1* and *IGFBP3* has been demonstrated to facilitate carcinogenesis and have an effect on cancer prognosis^10^. Chuang *et al*. found, using a combination of cDNA microarrays, western blot, and immunohistochemistry, that *IGFBP3* was a marker for clear cell RCC and that increased *IGFBP3* expression was associated with a higher Fuhrman grade^12^. In addition, it was reported that serum insulin-like growth factor-1 was an independent predictor of prognosis in patients with RCC^13^.

Findings from several studies have consistently found that genetic polymorphisms in *IGF1* and/or *IGFBP3* are associated with the risk or prognosis of various cancers, including colorectal cancer^14^, lung cancer^15^, prostate cancer^16^, and breast cancer^17^. However, the number of studies that have tested polymorphisms at the *IGF1* or *IGFBP3* locus for evidence of association with RCC has been sparse. A polymorphism in *IGFBP3* (rs2854744) was found to be a risk factor for RCC; however, the study had a small sample size consisting of 158 patients and 316 controls^18^. In light of the critical role of *IGF1* and *IGFBP3* in RCC, it is possible that genetic variants from these two genes will have an effect on the risk and/or prognosis of RCC. Therefore, in our present study, we selected nine potentially functional polymorphisms in *IGF1* (rs6214, rs6218, rs35767, rs5742612, and rs5742714) and *IGFBP3* (rs2132572, rs2854744, rs2854746 and rs282734), and evaluated their association with risk and prognosis of RCC in a two-stage case-control study.

## Materials and Methods

### Study population

This ongoing study was started in May 2004 and was approved by the institutional review board of Nanjing Medical University. The details of the inclusion criteria were described previously^6^,^19^. In brief, all subjects were genetically unrelated ethnic Han Chinese individuals. Those individuals who received chemotherapy or radiotherapy before surgery, or had a different type of cancer, were excluded from the present study. The control subjects were genetically unrelated to the patients and they had no individual history of cancer. The first (original) set of patient and control cohorts tested in the study included 355 patients and 362 cancer-free controls that were recruited from May 2004 to October 2009. The patients in this set were followed up prospectively every 6 months from the time of enrollment until death or until last time of follow-up. Of this cohort, 41 patients (11.5%) were excluded because of a lack of adequate information for follow-up, and 3 additional cases (0.8%) were excluded due to low DNA quality. The second, validation set comprised of 672 RCC cases and 703 controls. In the both sets, controls were frequency-matched to cases by age (±5 years) and sex. Each participant donated 5 ml venous blood collected in an EDTA tube after providing written informed consent. This study was approved by the Local Ethics Committees of the First Affiliated Hospital with Nanjing Medical University and were carried out in accordance with the approved guidelines. At recruitment, the written informed consent was obtained from each subject.

### SNP selection and genotyping

Polymorphisms in *IGF1* and *IGFBP3* were selected using genotype data from unrelated Han Chinese individuals in Beijing from the HapMap database (HapMap Data Rel 24/phaseII Nov08, on NCBI B36 assembly, dbSNP b126). Polymorphisms were considered from 2 kb upstream of the *IGF1* and *IGFBP3* locus to 2 kb downstream. We identified potentially functional polymorphisms according to the following criteria: (1) located in the 5′ flanking region, the 5′ untranslated region (UTR), the 3′-UTR, or the coding region causing an amino acid change and (2) a minor allele frequency (MAF) of greater than 5% in the Chinese population. Five polymorphisms in *IGFI* (rs6214, rs6218, rs35767, rs5742612, and rs5742714) and four in *IGFBP3* (rs2132572, rs2854744, rs2854746, and rs282734) were selected. Genotyping was performed using the TaqMan SNP genotyping method, as we previously described^20^.

### Quantitative measurement of IGF1 and IGFBP3 serum levels

Serum samples were available from 100 RCC cases and 100 controls. Collected serum was stored to clot for 30 minutes at 4 °C before centrifugation at 2000 rpm for 10 minutes at 4 °C. Serum was isolated and stored at −80 °C before use. IGF1 and IGFBP3 concentrations were measured using a sandwich enzyme immunoassay (Quantikine immunoassay kit, R&D Systems Inc, Minneapolis, MN, USA) and calculated using a standard curve according to the manufacturer’s instructions.

### Analysis of IGF1 mRNA expression

Forty-six surgically removed paratumor renal samples were used to measure *IGF1* mRNA levels *in vivo*. All tissue samples were stored in liquid nitrogen immediately following collection. RNA extraction and cDNA preparation was described previously^21^. *IGF1* mRNA was measured using quantitative real-time reverse transcription (RT)-PCR on an ABI Prism 7900 sequence detection system (Applied Biosystems, Foster City, CA, USA). The *ACTB* gene was used as an internal reference. The primers used for *IGF1* were 5′-GCTCTTCAGTTCGTGTGTGGA-3′ (sense) and 5′-GCCTCCTTAGATCACAGCTCC-3′ (antisense). Amplification conditions were as described previously^21^.

### Cell lines

HEK-293 and renal cell adenocarcinoma cell line 786-o were provided by Dr. Z. Zhang (Department of Molecular and Genetic Toxicology, School of Public Health, Nanjing Medical University, Nanjing, China) and were used as previously reported^22^.

### Construction of *IGF1* 3′-UTR reporter plasmids

*IGF1* 3′-UTRs (bps) containing either the G allele or C allele of rs5742714 were inserted into the *XbaI* site of pGL3 promoter vectors (Genscript, Nanjing, China). The accuracy of the constructed plasmids was verified by DNA sequencing.

### Dual-luciferase reporter assay

MiRNASNP v2.0 analysis was used to discover a seven-nucleotide complementary sequence between miR-580 and the IGF1 3′UTR^23^. A wild type (wt) IGF1 3′UTR sequence (5′-ACTTACAGACACTGAATTAATTTCCCCTGCTACTTTGAAACCAG AAAATATGACTGGCCATTCGTTACATCTGTCTTAGTTGAAAAGCATATTTTTTATTAAATTAATTCTGATTGTATTT GAAATTATTATTCAATTCACTTATGGCAGAGGAATATCAATCCTAATGACTTCTAAAA ATGTAACTAA TTGAATCATT ATCTTACATT-3′) in addition to a mutant type (mut) IGF1 3′UTR sequence with a rs5742714 different miR-580-binding sequence.

HEK-293 and 786-o cells were used for cell transfection and luciferase assays. Cells were seeded into culture medium-containing (100 μL/well) 96-well plates at a cell concentration of 1.5 × 10^4^ cells/well, followed by 24-hour incubation (37 °C, 100% humidity, 5% CO_2_). The cells were allocated into four groups: Group A, which were to transfeced a non-targeting negative control RNA with pGL3 IGF-G allele; Group B, which were to transfeced a non-targeting negative control RNA with pGL3 IGF-C allele;Group C, which were transfected with miR-580 mimics and pGL3-IGF1-G; and Group D, which were transfected with miR-580 mimics and pGL3-IGF1-C, using Lipofectamine 2000 (Invitrogen Corp, CA, USA). As an internal standard, all plasmids were cotransfected with pRL-TK, which contained the *Renilla* luciferase gene. After transfection for 48 hours, luciferase activity was measured with a Dual-Luciferase Reporter Assay System (Promega). Independent triplicate experiments were performed for each plasmid construct.

### Statistical Analysis

Differences in the distribution of demographic characteristics, selected variables, and frequencies of genotypes between cases and controls were evaluated using the Student’s t-test (for continuous variables) or the chi-square test (for categorical variables). Differences in serum levels of IGF1 and IGFBP3 among different genetic groups were evaluated using the Student’s t-test or one-way analysis of variance (ANOVA). Allele frequencies of each polymorphism were tested for deviations from Hardy-Weinberg equilibrium using a chi-square goodness-of-fit test before analysis. Associations between polymorphisms and risk of RCC were estimated using computing odds ratios and 95% confidence intervals (CIs) from unconditional logistic regression analysis with adjustment for possible confounders. Survival time was calculated from the date of RCC diagnosis to the date of death or last follow-up. Different survival times according to demographic characteristics, clinical features, and *IGF1* or *IGFBP3* polymorphisms were estimated using the Kaplan–Meier method and compared using the Log-rank test; univariate or multivariate Cox regression analysis was performed to determine predictive factors of RCC survival by estimating the hazard ratios (HRs). Differences in luciferase reporter gene expression among different promoter constructs as well as mRNA levels from tissues with different genotypes were evaluated using Student’s t-test or ANOVA. All analyses were performed using SAS 9.1.3 (SAS Institute, Cary, NC) with two-sided *P* values. A *P* value of less than 0.05 was considered statistically significant.

## Results

### Characteristics of RCC patients and controls

Frequency distributions of selected characteristics of the cohorts from both the original and validation sets are presented in Table 1. There were no significant differences between cases and controls with regard to age, gender, and drinking status in both sets of cohorts (all *P* > 0.05). However, there were more smokers, patients with hypertension, and diabetics among the group of cases compared to that of controls. We also found that the percentage of patients from the original cohort with stage I, II, III, and IV disease was 65.3%, 19.5%, 7.1%, and 8.1%, respectively, while patients from the validation set showed percentages of 21.6%, 51.1%, 20.7%, and 6.5%, respectively.

### Association of *IGF1* and *IGFBP3* polymorphisms with risk of RCC

The associations between these polymorphisms and risk of RCC in the best genetic model are presented in Table 2, and detailed genotype distributions of the polymorphisms in cases and controls are shown in Supplement Table 1. Genotype frequencies of all these nine polymorphisms in controls conformed to Hardy-Weinberg equilibrium (*P* > 0.05), with the exception of rs6218 in *IGF1*, which was removed from further analysis. In the test set, we found that the *IGF1* rs5742714 and rs6214 polymorphisms were significantly associated with a decreased risk of RCC (OR = 0.66, 95% CI = 0.48–0.92 and OR = 0.65, 95% CI = 0.45–0.86 for rs5742714 and rs6214, respectively). However, in the validation set, only the association between the *IGF1* rs5742714 polymorphism and decreased risk of RCC was verified (OR = 0.79, 95% CI = 0.63–0.98, GC/CC *vs.* GG). When patient cohorts were combined from both sets, as shown in Table 2, we found that affected individuals with either the rs5742714 GC or CC genotype had a significantly decreased risk of RCC (OR = 0.82, 95% CI = 0.68–0.98) compared to individuals with the rs5742714 GG genotype. No significant evidence of association was found between any of the other polymorphisms from *IGF1* or *IGFBP3* and risk of RCC.

### Effects of *IGF1* and *IGFBP3* polymorphisms on survival of RCC patients

**Figure 1:**
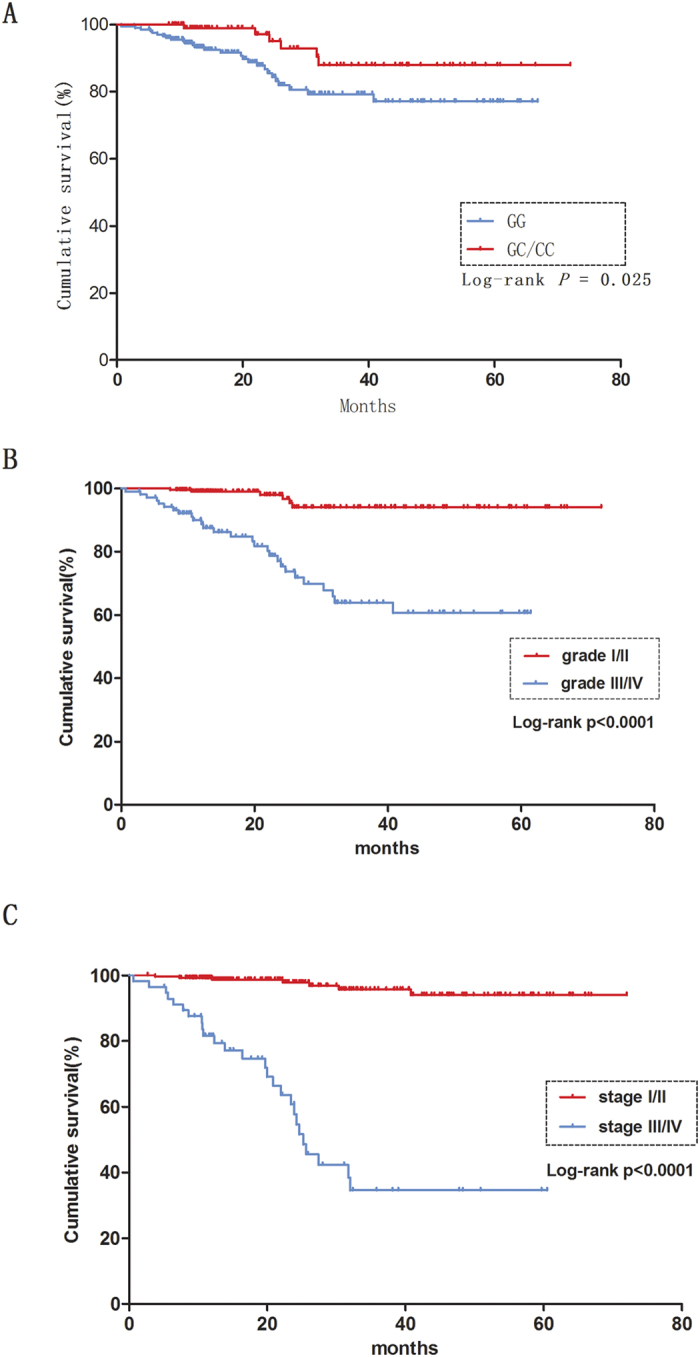
Kaplan-Meier survival curves showing RCC overall survival according to: (**A**) *IGF1* rs5742714 (GC/CC vs. GG), (**B**) tumor grade (I + II and III + IV), and (**C**) clinic stage (I + II and III + IV).

We then investigated patients from the first set, who had available follow-up information, for evidence of association between these polymorphisms and survival of RCC patients using the Log-rank test and Cox regression analysis. We found that the *IGF1* rs5742714 polymorphism was significantly associated with patient survival. As shown in Table 3 and Fig. 1A, compared with patients with the GG genotype, those harboring the GC or GC/CC genotypes showed improved RCC survival (HR = 0.36, 95% CI = 0.14–0.94 and HR = 0.36, 95% CI = 0.14–0.93, respectively; Log-rank *P* = 0.031 and *P* = 0.025, respectively). However, no significant evidence of association was found between the other polymorphisms tested and RCC survival (data not shown).

### Cox proportional hazard analysis for overall survival of RCC

We further performed a univariate and multivariate Cox proportional hazard analysis for survival of RCC patients. As shown in Table 4 and Fig. 1, clinical stage, tumor grade, and IGF1 rs5742714 (GC/CC *vs.* GG) were associated with RCC survival in univariate analysis; a multivariate analysis found that clinical stage was the best prognostic factor for RCC survival, followed by tumor grade (*P* < 0.001, HR = 20.28; 95% CI = 8.78–46.87 and *P* = 0.002, HR = 4.24; 95% CI = 1.70–10.6, respectively). Interestingly, *IGF1* rs5742714 (GC/CC *vs.* GG) was also an independent predictor of RCC survival (*P* = 0.035, HR = 0.36; 95% CI = 0.14–0.93).

### Effect of *IGF1* rs5742714 genotype on *IGF1* expression

**Figure 2:**
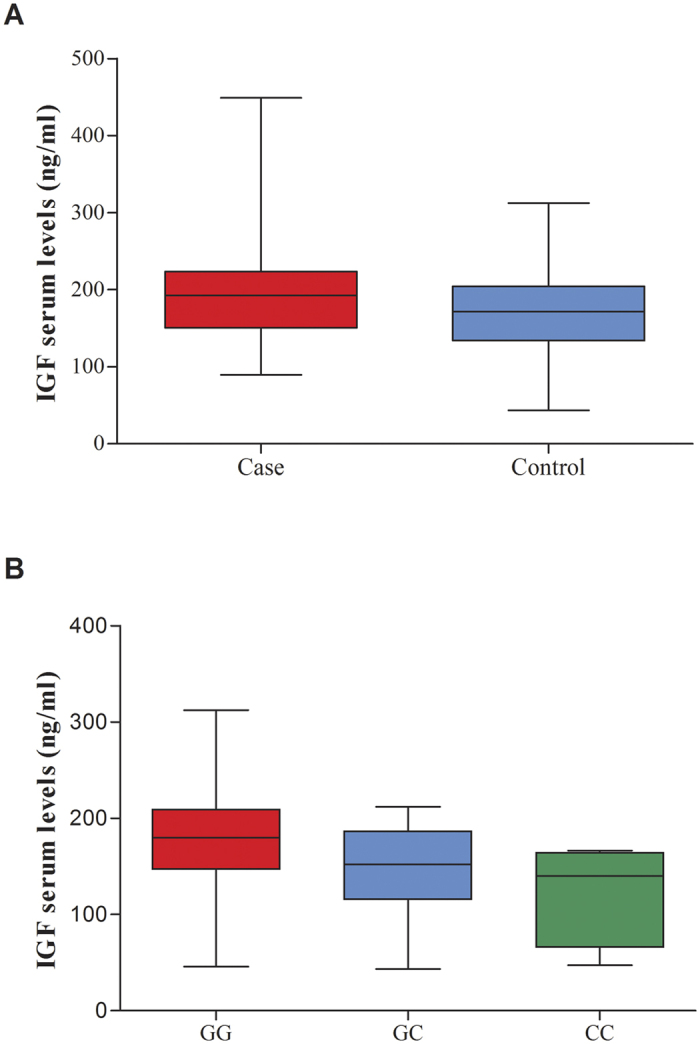
Serum levels of *IGF1* in cases and controls. (**A**) Distribution of serum IGF1 levels in 100 cases and 100 controls. The mean level of serum IGF1 in cases was significantly higher than that in controls (*P* = 0.005); (**B**) Distribution of serum IGF1 levels in controls with different *IGF1* rs5742714 genotypes. The number of subjects with a GG, GC, and CC genotype was 70, 26, and 4, respectively. The mean level of serum *IGF1* in *IGF1* rs5742714 GC and CC groups were significantly lower than that found in the GG groups (*P* = 0.018 and *P* = 0.049, respectively).

Next, we investigated IGF1 serum levels in RCC patients and controls, and tested for association between *IGF1* rs5742714 genotypes and serum levels. As shown in Fig. 2A, the median serum level of IGF1 was significantly higher in cases compared to that in controls (*P* = 0.005). In addition, and as shown in Fig. 2B, individuals with either the GC or CC genotype had significantly lower serum IGF1 levels than individuals carrying the rs5742714 GG genotype (*P* = 0.018 and *P* = 0.049, respectively).

### Functional characterization of *IGF1* rs5742714 polymorphism

**Figure 3:**
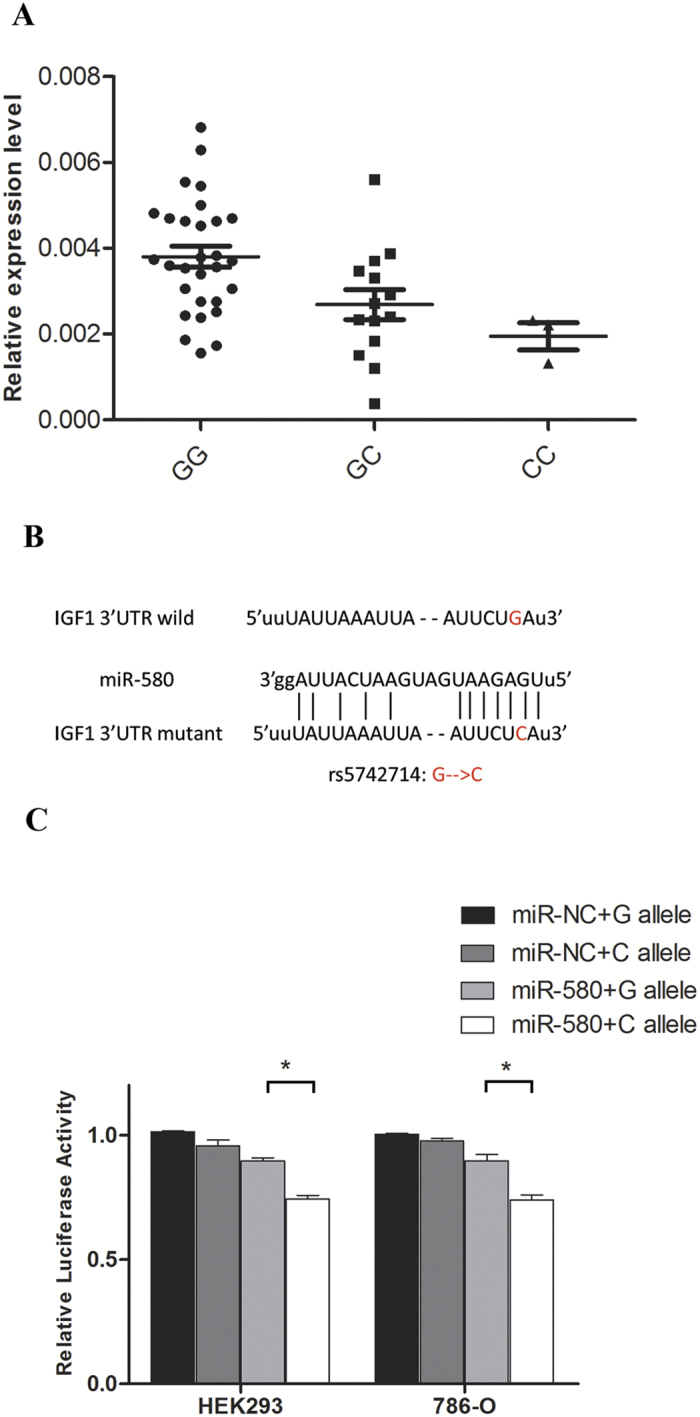
Effect of *IGF1* rs5742714 polymorphism on *IGF1* expression. (**A**) Association between *IGF1* expression in renal tissues and *IGF1* rs5742714 genotypes. Compared to individuals with the rs5742714 GG genotype, individuals with the rs5742714 GC or CC genotype were associated with significantly decreased *IGF1* mRNA levels (*P* = 0.013 and *P* = 0.017, respectively); (**B**) Bioinformatics analysis predicted a binding site between miR-580 and *IGF1*; (**C**) Transient transfection of reporter and mimics into HEK293 and 786-o cell lines. Luciferase activity was measured using a Dual-Luciferase Reporter Assay System (Promega). Values are mean ± SD from more than three separate experiments that were each performed in triplicate. *Indicates a significant difference (*P* < 0.05). There is significant difference between Group C, which were transfected with miR-580 mimics and pGL3-IGF1-G and Group D, which were transfected with miR-580 mimics and pGL3-IGF1-C.

To further explore the potential functionality of rs5742714, we investigated the effect of this polymorphism on *IGF1* expression in renal tissues using real-time quantitative PCR. As shown in Fig. 3A, individuals with the GG or GC genotype had lower levels of *IGF1* expression than individuals with the GG genotype, (*P* = 0.013 and *P* = 0.017, respectively). As predicted using a bioinformatics model^23^, the change from a G allele to a C allele at rs5742714 may create a microRNA (miRNA) binding site for hsa-mir-580. To test this finding, we constructed different pGL3 vectors which included the allele-specific binding sequences (Fig. 3B), and co-transfected with miR-508 mimics as well as controls in HEK-293 and 786-o cell lines. As shown in Fig. 3C, we observed significantly greater luciferase activity with the reporter containing the rs5742714 G allele than that containing the rs5742714 C allele. These findings indicate that the rs5742714 C allele may result in inhibition of *IGF1* expression through creation of a miRNA binding site.

## Discussion

In the present study, we investigated whether there was evidence of association of polymorphisms from the *IGF1* and *IGFBP3* loci with RCC development and survival in a Chinese population. We found that the functional polymorphism rs5742714 in the 3′-UTR of the *IGF1* gene was associated with decreased risk of RCC. We also found that the *IGF1* rs5742714 polymorphism was an independent prognostic predictor of RCC survival, along with clinical stage and tumor grade, in multivariate analysis. The functional role of rs5742714 was further shown by its effect on IGF1 serum levels. To our knowledge, this is the first study to demonstrate a role of *IGF1* rs5742714 in the etiology and prognosis of RCC.

*IGF1* and *IGFBP3* have been implicated in the development and progression of various human cancers^24^, including RCC^13^,^25^,^26^. In the present study, we found that patients with RCC had higher IGF1 serum concentrations compared to controls. Genetic variations in the two genes have been extensively tested and associated with the development and progression of several types of cancer. Previous studies found that the *IGF1* rs5742714 polymorphism was associated with cancer risk and prognosis. Nakao *et al*. found in a Japanese population that this polymorphism may have an effect on the development of pancreatic cancer in combination with obesity^27^. In another study performed in a Chinese population, Zhang *et al*. demonstrated that *IGF1* rs5742714 was a genetic modifier for NSCLC prognosis, especially among patients who underwent surgery^15^. Similar to findings from our study, they found that patients with either the rs5742714 GC or CC genotype had a favorable overall survival (HR = 0.77, 95% CI = 0.60–0.99).

Considering the position of the rs5742714 polymorphism in the 3′-UTR of the *IGF1* gene, it was predicted that the rs5742714 variant might have an effect on *IGF1* expression by altering *IGF1* mRNA stability and its binding ability to microRNAs (miRNAs). Interestingly, as predicted using a bioinformatics model^23^, substitution of the rs5742714 G allele by the C allele may create an miRNA binding site for hsa-mir-580, which was demonstrated to act as a tumor inhibitor in breast cancer, through negatively regulating *TWIST1* expression^28^. We hypothesize that the rs5742714 C allele results in miRNA binding to the region, and subsequently, down-regulation of *IGF1* expression by either mRNA cleavage or translational repression. This is in agreement with our findings from this study in which individuals with the rs5742714 GC or CC genotype showed decreased IGF1 serum levels compared to individuals with the rs5742714 GG genotype. Given the role of *IGF1* in cancer development and progression, reduced levels of *IGF1* because of the rs5742714 genotype may decrease cancer susceptibility and inhibit progression, which may explain our findings in the case-control study. However, the exact mechanism underlying the functional effects of this polymorphism on *IGF1* expression requires further investigation.

The *IGFBP3* rs2854744 polymorphism has been studied in different cancers such as esophageal squamous cell carcinoma^29^, breast cancer^30^, colorectal cancer^31^, and urinary bladder cancer^32^. The polymorphism is located in the gene promoter and may have an effect on the binding affinity of transcription factors to the promoter, which would lead to changes in gene expression. There are several lines of evidence that suggest that the *IGFBP3* rs2854744 A allele may enhance promoter activity and may be associated with higher levels of circulating IGFBP3^33^,^34^. However, in our study, we did not find evidence for association between this polymorphism and risk or prognosis of RCC. There was also no evidence of association between IGFBP3 serum levels and rs2854744 genotype at the *IGFBP3* locus. It should be noted that this polymorphism was previously found to be associated with the development of clear cell RCC in an Iranian population^18^; however, the sample size of the study was relatively small (158 patients and 316 controls vs. 1027 cases and 1094 controls in our study). In addition, other factors such as different genetic backgrounds may also account for the disparity between our studies. For instance, the minor allele frequency of *IGFBP3* rs2854744 in their study differed dramatically from that found in ours (A allele 40.8% vs. C allele 24.1%).

In conclusion, this is the first study demonstrating a role of the *IGF1* rs5742714 polymorphism in RCC susceptibility and prognosis in a Chinese cohort. In addition, the findings from the present study also highlight the putatively functional effect of this variant on *IGF1* expression by changes mediated by miRNA regulation. Our large two-stage case-control study provided the statistical power to identify polymorphisms associated with risk of RCC; however, the number of patients available for survival analysis were limited and require independent confirmation. Therefore, further validation in a larger population and functional studies are required to understand the role of *IGF1* in RCC better.

## Additional Information

**How to cite this article**: Cao, Q. *et al*. Genetic variation in *IGF1* predicts renal cell carcinoma susceptibility and prognosis in Chinese population. *Sci. Rep.* **6**, 39014; doi: 10.1038/srep39014 (2016).

**Publisher's note:** Springer Nature remains neutral with regard to jurisdictional claims in published maps and institutional affiliations.

## Supplementary Material

Supplementary Table 1

## Figures and Tables

**Table 1:** Demographic and clinical features of RCC patients and control subjects in the discovery and validation sets.

Characteristics	Discovery set (N, %)		Validation set (N, %)		Combined set (N, %)		*P*^a^
	Case	Controls	Case	Controls	Case	Controls	
Overall	355	362	672	732	1027	1094	
Age (mean ± SD)	57.1 ± 11.8	56.4 ± 13.1	56.5 ± 12.2	57.5 ± 11.4	56.7 ± 12.1	57.1 ± 12.1	0.522
Gender							
Male	228 (64.2)	212 (58.6)	424 (63.1)	492 (66.1)	652 (63.5)	704 (64.4)	0.678
Female	127 (35.7)	150 (41.4)	248 (36.9)	240 (33.9)	375 (36.3)	390 (35.6)	
Smoking status							
Never	220 (62.0)	242 (66.9)	427 (63.5)	501 (68.4)	647 (63.0)	743 (67.9)	**0.017**
Former	135 (38.0)	120 (33.1)	245 (36.5)	231 (31.6)	380 (37.0)	351 (32.1)	
Drinking status							
Never	249 (70.1)	248 (68.5)	501 (74.6)	559 (76.4)	750 (73.0)	807 (73.8)	0.701
Ever	106 (29.9)	114 (31.5)	171 (25.4)	173 (23.6)	277 (27.0)	287 (26.2)	
Hypertension							
No	220 (62.0)	255 (70.4)	406 (60.4)	555 (75.8)	626 (70.0)	810 (74.0)	**<0.001**
Yes	135 (38.0)	107 (29.6)	266 (39.6)	177 (24.2)	401 (30.0)	284 (26.0)	
Diabetes							
No	310 (87.3)	345 (95.3)	585 (87.0)	682 (93.2)	895 (87.2)	1027 (93.9)	**<0.001**
Yes	45 (12.7)	17 (4.7)	87 (13.0)	50 (6.8)	132 (12.8)	67 (6.1)	
Clinical stage							
I	227 (63.9)		444 (66.1)		671 (65.3)		
II	63 (17.7)		137 (20.4)		200 (19.5)		
III	21 (5.9)		52 (7.7)		73 (7.1)		
IV	44 (12.4)		39 (5.8)		83 (8.1)		
Grade							
I	68 (19.2)		155 (23.1)		222 (21.6)		
II	170 (47.9)		355 (52.8)		525 (51.1)		
III	84 (23.7)		128 (19.1)		213 (20.7)		
IV	33 (9.3)		34 (5.1)		67 (6.5)		
Histology							
Clear cell	301 (84.8)		558 (83.0)		860 (83.7)		
Papilarry	8 (2.3)		29 (4.3)		36 (3.5)		
Chromophobe	17 (4.8)		38 (5.7)		55 (5.4)		

^a^Student’s t-test for age distributions between cases and controls; two-sided χ^2^-test for others selected variables between cases and controls.

**Table 2:** Genetic associations between polymorphisms in *IGF1*/*IGFBP3* and risk of renal cell carcinoma.

The best genetic model*								
Stages	SNPs	Location	Cases†	Controls†	MAF	*P* for HWE	*P*	OR
Test set								
*IGF1*	rs6214 G > A	3′ UTR	90/168/97	109/182/71	0.447	0.750	**0.015**	**0.65 (0.45–0.86)**
	rs6218 T > C	3′ UTR	207/125/23	207/143/12	0.231	0.032	0.760	0.94 (0.69**–**1.29)
	rs35767 C > T	5′ near gene	152/152/51	165/160/37	0.323	0.885	0.456	1.13 (0.82**–**1.55)
	rs5742612 T > C	5′ near gene	194/140/21	209/137/17	0.236	0.360	0.448	1.26 (0.97**–**1.86)
	rs5742714 G > C	3′ UTR	249/99/7	225/114/23	0.221	0.104	**0.024**	**0.66 (0.48–0.92)**
*IGFBP3*	rs2132572 G > A	5′ near gene	224/116/15	240/111/11	0.184	0.670	0.370	1.07 (0.77**–**1.47)
	rs2854744 A > C	5′ near gene	208/114/33	198/142/22	0.257	0.602	0.293	0.82 (0.60**–**1.12)
	rs2854746 C > G	Missense	217/108/30	228/118/16	0.207	0.883	0.608	1.12 (0.82**–**1.55)
	rs282734 A > C	Missense	323/30/2	325/36/1	0.052	0.997	0.584	0.77 (0.46**–**1.29)
Validation								
*IGF1*	rs5742714	3′ UTR	464/180/28	466/241/25	0.199	0.363	**0.040**	**0.79 (0.63–0.98)**
	rs6214 G > A	3′ UTR	193/326/153	192/368/172	0.486	0.866	0.239	0.86 (0.68**–**1.10)
Combined								
*IGF1*	rs5742714	3′ UTR	713/279/35	691/355/48	0.206	0.779	**0.002**	**0.82 (0.68–0.98)**

OR, odds ratio; HWE (Hardy–Weinberg equilibrium) test among controls; Values in bold indicate they are statistically different.

^*^Logistic regression model with adjustment for age, sex, BMI, smoking status, drinking status, hypertension, diabetes and family history of cancer; detail information on the results the SNPs were demonstrated in Supplemental Table 1. All the best genetic model of the SNPs was recessive model, except for rs6214, of which the best genetic model was dominant model.

^†^Major homozygote/heterozygote/minor homozygote between cases and controls.

**Table 3:** Associations between the *IGF1* rs5742714 polymorphism and RCC patients’ survival.

*IGF1* rs5742714	Patients (N = 311)	Deaths (N = 33)	5-yr survival* (%)	Log-Rank *P*	HR (95% CI)†
Codominant model					
^ ^GG	205	27	77.1%		1.00 (reference)
^ ^GC	93	5	88.9%	**0.031**	**0.36 (0.14–0.94)**
^ ^CC	13	1	85.7%	0.440	0.47 (0.06**–**3.51)
Additive model				**0.046**	**0.45 (0.21–0.99)**
Dominant model					
^ ^GG	205	27	77.1%	**0.025**	1.00 (reference)
^ ^GC/CC	106	6	88.0%		**0.36 (0.14–0.93)**

HR: hazards ratio; CI: confidence interval.

^*^Proportion of survival derived from Kaplan-Meier analysis.

^†^Adjusted for age, gender, smoking, drinking status, diabetes and hypertension as well as tumor grade and clinic stage.

**Table 4:** Univariate and multivariate Cox proportional hazard analysis of death risk in patients with RCC.

Parameters	Univariate		Multivariate†	
	HR (95% CI)*	*P* value	HR (95% CI)^a^	*P* value
Age (≤56 vs. >56)	1.68 (0.83**–**3.37)	0.147		
Gender (female vs. male)	0.89 (0.44**–**1.82)	0.758		
Smoking status (never vs. ever)	0.97 (0.47**–**2.00)	0.929		
Drinking status (never vs. ever)	1.33 (0.63**–**2.80)	0.458		
Diabetes (no vs. yes)	0.66 (0.20**–**2.16)	0.493		
Hypertension (no vs. yes)	1.53 (0.77**–**3.05)	0.222		
Tumor grade (III/IV vs. I/II)	**8.88 (3.66–21.52)**	**<0.001**	4.24 (1.70**–**10.6)	0.002
Clinical stage (III/IV vs. I/II)	**20.28 (8.78–46.87)**	**<0.001**	15.8 (6.28**–**39.5	**<0.001**
*IGF* rs5742714 (GC/CC vs. GG)	**0.37 (0.16–0.91)**	**0.031**	0.36 (0.14**–**0.93)	0.035

^*^HR: hazards ratio; CI: confidence interval.

^†^In this multivariate analysis age, gender, smoking, drinking status, diabetes, hypertension, tumor stage, clinic stage and the number of variant alleles were included.
