# Supplementary material for: Genetic variation in IGF1 predicts renal cell carcinoma susceptibility and prognosis in Chinese population
Source: Sci Rep. 2016 Dec 15;6:39014. doi: 10.1038/srep39014 (PMC5157037; doi:10.1038/srep39014)
Supplement: Supplementary Table 1 [file srep39014-s1.doc]

**Genetic variation in *IGF1* predicts renal cell carcinoma susceptibility and prognosis in Chinese population**

Qiang Cao^1^, Chao Liang ^1^, Jianxin Xue^1^, Pu Li ^1^, Jie Li^1^, Meilin Wang ^2^, Zhengdong Zhang ^2^, Chao Qin^1^, Qiang Lu^1^, Lixin Hua^1^, Pengfei Shao^1^, *****, Zengjun Wang^1^, *****

**Supplemental table 1 Genotypic distribution of the selected polymorphism in cases and controls**

* Adjusted for age, gender, smoking, drinking status, diabetes and hypertension as well as tumor grade and clinic stage.

| Genotypes | Original set | | |  | Validation set | | |  | Combined set | | | |
| --- | --- | --- | --- | --- | --- | --- | --- | --- | --- | --- | --- | --- |
|  | Case | Control | *P* |  | Case | Control | *P* |  | Case | Control | *P* | OR * |
| *IGF1* rs5742714 |  |  |  |  |  |  |  |  |  |  |  |  |
| GG | 249 | 225 |  |  | 464 | 466 |  |  | 713 | 691 |  | 1.00 (reference) |
| GC | 99 | 114 | 0.142 |  | 180 | 241 | **0.015** |  | 279 | 355 | **0.005** | **0.82 (0.67-0.99)** |
| CC | 7 | 23 | **0.002** |  | 28 | 25 | 0.677 |  | 35 | 48 | 0.127 | 0.79 (0.50-1.27) |
| GC/CC | 106 | 137 | **0.024** |  | 208 | 266 | **0.033** |  | 314 | 403 | **0.002** | **0.82 (0.68-0.98)** |
| *IGF1* rs35767 |  |  |  |  |  |  |  |  |  |  |  |  |
| CC | 152 | 165 |  |  | 314 | 311 |  |  | 466 | 476 |  | 1.00 (reference) |
| CT | 152 | 160 | 0.847 |  | 283 | 342 | 0.079 |  | 435 | 502 | 0.187 | 0.92 (0.76-1.11) |
| TT | 51 | 37 | 0.097 |  | 75 | 79 | 0.732 |  | 126 | 116 | 0.471 | 1.06 (0.80-1.43) |
| CT/TT | 203 | 197 | 0.456 |  | 358 | 421 | 0.110 |  | 561 | 618 | 0.388 | 0.95 (0.80-1.40) |
| *IGF1* rs5742612 |  |  |  |  |  |  |  |  |  |  |  |  |
| TT | 194 | 208 |  |  | 358 | 388 |  |  | 552 | 596 |  | 1.00 (reference) |
| TC | 140 | 137 | 0.559 |  | 249 | 280 | 0.746 |  | 389 | 417 | 0.938 | 1.02 (0.85-1.24) |
| CC | 21 | 17 | 0.409 |  | 65 | 64 | 0.615 |  | 86 | 81 | 0.410 | 1.04 (0.75-1.47) |
| TC/CC | 161 | 154 | 0.448 |  | 314 | 344 | 0.920 |  | 475 | 498 | 0.736 | 1.03 (0.86-1.23) |
| *IGF1* rs6218 |  |  |  |  |  |  |  |  |  |  |  |  |
| TT | 207 | 207 |  |  |  |  |  |  |  |  |  |  |
| TC | 125 | 143 | 0.391 |  |  |  |  |  |  |  |  |  |
| CC | 23 | 12 | 0.074 |  |  |  |  |  |  |  |  |  |
| TC/CC | 148 | 155 | 0.760 |  |  |  |  |  |  |  |  |  |
| *IGF1* rs6214 |  |  |  |  |  |  |  |  |  |  |  |  |
| GG | 90 | 109 |  |  | 193 | 192 |  |  | 283 | 301 |  | 1.00 (reference) |
| GA | 168 | 182 | 0.531 |  | 326 | 368 | 0.320 |  | 494 | 550 | 0.658 | 0.93 (0.75-1.15) |
| AA | 97 | 71 | 0.039 |  | 153 | 172 | 0.437 |  | 250 | 243 | 0.462 | 1.08 (0.84-1.40) |
| GA/AA | 265 | 253 | 0.155 |  | 479 | 540 | 0.296 |  | 744 | 793 | 0.983 | 0.98 (0.80-1.20) |
| GG/GA | 258 | 291 |  |  | 519 | 560 | 0.746 |  | 777 | 851 |  | 1.00 (reference) |
| AA | 97 | 71 | **0.015** |  | 153 | 172 |  |  | 250 | 243 | 0.246 | 1.13 (0.92-1.40) |
| *IGFBP3* rs9282734 |  |  |  |  |  |  |  |  |  |  |  |  |
| AA | 323 | 325 |  |  | 604 | 667 |  |  | 927 | 992 |  | 1.00 (reference) |
| AC | 30 | 36 | 0.491 |  | 63 | 64 | 0.654 |  | 93 | 100 | 0.975 | 1.00 (0.73-1.36) |
| CC | 2 | 1 | 0.561 |  | 5 | 1 | 0.080 |  | 7 | 2 | 0.078 | 3.71 (0.76-18.10) |
| AC/CC | 32 | 37 | 0.584 |  | 68 | 65 | 0.428 |  | 100 | 102 | 0.746 | 1.06 (0.78-1.43) |
| *IGFBP3* rs2132572 |  |  |  |  |  |  |  |  |  |  |  |  |
| GG | 224 | 240 |  |  | 429 | 449 |  |  | 653 | 689 |  | 1.00 (reference) |
| GA | 116 | 111 | 0.485 |  | 217 | 255 | 0.311 |  | 333 | 366 | 0.662 | 0.93 (0.76-1.12) |
| AA | 15 | 11 | 0.350 |  | 26 | 28 | 0.919 |  | 41 | 39 | 0.652 | 1.11 (0.68-1.80) |
| GA/AA | 131 | 122 | 0.370 |  | 243 | 283 | 0.334 |  | 374 | 405 | 0.773 | 0.94 (0.78-1.13) |
| *IGFBP3* rs2854746 |  |  |  |  |  |  |  |  |  |  |  |  |
| CC | 217 | 228 |  |  | 389 | 441 |  |  | 606 | 669 |  | 1.00 (reference) |
| CG | 108 | 118 | 0.811 |  | 248 | 243 | 0.201 |  | 356 | 361 | 0.363 | 1.07 (0.89-1.30) |
| GG | 30 | 16 | 0.034 |  | 35 | 48 | 0.413 |  | 65 | 64 | 0.536 | 1.02 (0.70-1.49) |
| CG/GG | 138 | 134 | 0.608 |  | 283 | 291 | 0.369 |  | 421 | 425 | 0.313 | 1.07 (0.89-1.28) |
| *IGFBP3* rs2854744 |  |  |  |  |  |  |  |  |  |  |  |  |
| AA | 208 | 198 |  |  | 392 | 428 |  |  | 600 | 626 |  | 1.00 (reference) |
| AC | 114 | 142 | 0.093 |  | 245 | 267 | 0.987 |  | 359 | 409 | 0.340 | 0.93 (0.77-1.12) |
| CC | 33 | 22 | 0.222 |  | 35 | 37 | 0.896 |  | 68 | 59 | 0.323 | 1.24 (0.85-1.82) |
| AC/CC | 147 | 164 | 0.293 |  | 280 | 304 | 0.959 |  | 427 | 468 | 0.576 | 0.97 (0.81-1.16) |
